# Does providing feedback and guidance on sleep perceptions using sleep wearables improve insomnia? Findings from “Novel Insomnia Treatment Experiment”: a randomized controlled trial

**DOI:** 10.1093/sleep/zsad167

**Published:** 2023-06-09

**Authors:** Marie-Antoinette Spina, Thomas Andrillon, Nina Quin, Joshua F Wiley, Shantha M W Rajaratnam, Bei Bei

**Affiliations:** Turner Institute for Brain and Mental Health, School of Psychological Sciences, Faculty of Medicine, Nursing and Health Sciences, Monash University, Melbourne, VIC, Australia; School of Philosophical, Historical, and International Studies, Centre for Consciousness and Contemplative Studies, Monash University, Melbourne, VIC, Australia; Sorbonne Université, Institut du Cerveau - Paris Brain Institute - ICM, Inserm, CNRS, Paris, France; Turner Institute for Brain and Mental Health, School of Psychological Sciences, Faculty of Medicine, Nursing and Health Sciences, Monash University, Melbourne, VIC, Australia; Turner Institute for Brain and Mental Health, School of Psychological Sciences, Faculty of Medicine, Nursing and Health Sciences, Monash University, Melbourne, VIC, Australia; Peter MacCallum Cancer Centre, Melbourne, VIC, Australia; Turner Institute for Brain and Mental Health, School of Psychological Sciences, Faculty of Medicine, Nursing and Health Sciences, Monash University, Melbourne, VIC, Australia; Turner Institute for Brain and Mental Health, School of Psychological Sciences, Faculty of Medicine, Nursing and Health Sciences, Monash University, Melbourne, VIC, Australia

**Keywords:** Sleep, Sleep–wake estimation, Sleep–wake state discrepancy, Insomnia, Dreem, Fitbit

## Abstract

**Study Objectives:**

Insomnia is a disorder diagnosed based on self-reported sleep complaints. Differences between self-reported and sensor-based sleep parameters (sleep–wake state discrepancy) are common but not well-understood in individuals with insomnia. This two-arm, parallel-group, single-blind, superiority randomized-controlled trial examined whether monitoring sleep using wearable devices and providing support for interpretation of sensor-based sleep data improved insomnia symptoms or impacted sleep–wake state discrepancy.

**Methods:**

A total of 113 (age *M* = 47.53; SD = 14.37, 64.9% female) individuals with significant insomnia symptoms (Insomnia Severity Index(ISI) ≥10) from the community were randomized 1:1 (permuted block randomization) to receive 5 weeks (1) Intervention (*n* = 57): feedback about sensor-based sleep (Fitbit and EEG headband) with guidance for data interpretation and ongoing monitoring, and (2) Control (*n* = 56): sleep education and hygiene. Both groups received one individual session and two check-in calls. The ISI (primary outcome), sleep disturbance (SDis), sleep-related impairment (SRI), depression, and anxiety were assessed at baseline and post-intervention.

**Results:**

In total, 103 (91.2%) participants completed the study. Intention-to-treat multiple regression with multiple imputations showed that after controlling for baseline values, compared to the Control group (*n* = 51), the Intervention group (*n* = 52) had lower ISI (*p* = .011, *d* = 0.51) and SDis (*p* = .036, *d* = 0.42) post-intervention, but differences in SRI, depression, anxiety, and sleep–wake state discrepancy parameters (total sleep time, sleep onset latency, and wake after sleep onset) were not meaningful (*P*-values >.40).

**Conclusions:**

Providing feedback and guidance about sensor-based sleep parameters reduced insomnia severity and sleep disturbance but did not alter sleep–wake state discrepancy in individuals with insomnia more than sleep hygiene and education. The role of sleep wearable devices among individuals with insomnia requires further research.

**Clinical Trial Registration:**

The Novel Insomnia Treatment Experiment (NITE): the effectiveness of incorporating appropriate guidance for sleep wearables in users with insomnia. https://www.anzctr.org.au/Trial/Registration/TrialReview.aspx?id=378452, Australia New Zealand Clinical Trials Registry: ACTRN12619001636145.

## Graphical Abstract

**Figure:**
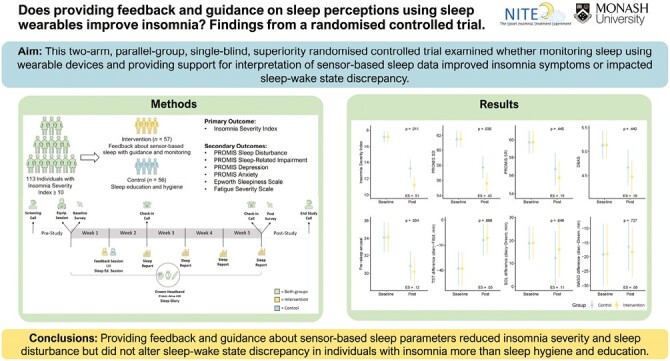

**Statement of Significance**

Numerous wearable devices measure sleep, but few provide feedback tailored to individuals with insomnia. Appropriate feedback is particularly important when sensor-based sleep measurements differ from individuals’ self-report assessment of sleep, which is frequent in insomnia. This adequately powered, randomized-controlled trial found that compared to sleep hygiene and education, providing monitoring and feedback about wearable sensor-based sleep with support for interpretation of sleep–wake state discrepancy improved insomnia symptoms and reduced sleep disturbance in individuals with insomnia. However, these effects were modest, and benefits to other aspects of sleep experiences were limited. These findings call for further research into better understanding sleep–wake state discrepancy in insomnia and into uncovering new approaches to supplement current insomnia treatment.

## Introduction

Insomnia involves difficulties with initiating sleep, maintaining sleep, and early morning awakenings [1]. Insomnia symptoms are experienced by 9%–30% of individuals in the general population [2–5] and are associated with daytime impairment such as fatigue, mood disturbances, and attention impairment [6].

Insomnia is diagnosed based on individual’s self-report (e.g. questionnaire, sleep diary, and clinical interview) [1, 6, 7]. Yet a variety of tools have been used to record sleep in insomnia, such as polysomnography (PSG) and actigraphy, which rely on different signals (e.g. EEG, physical activity) to infer sleep state [8]. Sleep information derived from different methods often differs. The difference between self-report and sensor-based methods has been termed sleep-state misperception [9], sleep misestimation [10], paradoxical insomnia [11, 12], or subjective-objective sleep discrepancy [13]. This manuscript uses the recently recommended phrase sleep–wake state discrepancy [8], which focuses on the differences between measures without inferring accuracy.

Sleep–wake state discrepancy is a common phenomenon observed in 9%–50% of individuals with insomnia [9, 14–17]. The most common direction of discrepancy in insomnia is self-reporting longer sleep onset latency (SOL; time to fall asleep), wake after sleep onset (WASO), and shorter total sleep time (TST), compared to sensor-based sleep estimates [18–21]. Sleep–wake state discrepancy is also observed in other sleep disorders including sleep apnea [22–24], periodic limb movement disorder [17], narcolepsy [25], as well as adults in the general population [26–28]. In some cases, however, individuals may self-report longer or better sleep, compared to sensor-based sleep measures (e.g. “reverse/positive sleep state misperception”) [29–31].

Sleep–wake state discrepancy is important to explore as it is linked to daytime functioning, self-reported happiness, social support [32, 33], and self-reported sleep (i.e. sleep efficiency, sleep quality, TST, and SOL) [34]. The underlying mechanisms of sleep–wake state discrepancy are unclear. Potential reasons include physiological factors (e.g. increase in fast wake-like rhythms during sleep, local modulations of sleep depth, and sensitivity to sleep fragmentation) [35–39], measurement factors (i.e. different sleep measures estimate sleep based on different signals), and cognitive factors (e.g. sleep-related worry, sleep-related cognitions, and selective sleep monitoring) [40]. Harvey’s cognitive model of insomnia suggests that individuals may become worried about sleep and daytime consequences of not getting enough sleep, which leads to behaviors such as increased sleep monitoring (e.g. clock checking, which may increase anxiety about being awake throughout the night) that further results in a perception of increased sleep deficits [41, 42].

Sleep–wake state discrepancy has been found to be modifiable. Downey and Bonnet (1992) [43] woke individuals with insomnia up 27 times per night to give them feedback about their PSG sleep; after feedback, participants reported an improved ability to fall asleep, and self-report and PSG-recorded estimates of sleep/wake were more aligned. Tang and Harvey (2004) [44] conducted a pilot study (*N* = 40), in which individuals with insomnia were randomly allocated into either “shown-discrepancy” or “non-demonstration” groups. The “shown-discrepancy” group were taught to read actigraphy data and calculate sleep–wake state discrepancy; subsequently, they self-reported shortened SOL and less anxiety and preoccupation about sleep compared to the “non-demonstration” group [44]. Tang and Harvey (2006) [45] expanded upon those findings with another experiment (*N* = 48), in which the control group were verbally told about sleep–wake state discrepancy without demonstration or calculation. Consistent with findings from the earlier study, those who completed the intervention had a greater reduction in self-reported sleep impairment, insomnia symptoms, and sleep-related anxiety and distress [45].

In recent years, with the increased use of consumer sleep wearable devices, individuals with insomnia are increasingly recording their own sleep data [46–49]. However, these devices do not typically provide guidance for how to interpret sensor-based recorded sleep data or inform users about the existence and meaning of discrepancy between devices’ measurements and individuals’ own perceived sleep.

This current study aims to examine whether providing individuals with insomnia feedback about sleep using wearable devices, along with support for appropriate interpretation of sleep–wake state discrepancy improves (1) symptoms of insomnia as the primary outcome, (2) sleep disturbance and sleep-related impairment (SRI)s, sleepiness, fatigue, symptoms of depression and anxiety as secondary outcomes, as well as (3) sleep–wake state discrepancy, dysfunctional beliefs and attitudes about sleep, and pre-sleep arousal (PSA) as exploratory outcomes; the latter two were explored due to their relevance to both insomnia and sleep–wake state discrepancy [50–53]. For example, beliefs such as “I need 8 hours of sleep to feel refreshed and function well during the day” could increase sleep monitoring and exacerbate the perception of poor sleep; this is consistent with the findings that individuals with higher sleep–wake state discrepancy have greater endorsement on such beliefs compared to “good” sleepers [21]. Similarly, higher PSA (e.g. worry about falling asleep, heart racing when attempting to sleep) may heighten perceived wakefulness and exacerbate the perception of poor sleep. Studies have found higher pre-sleep cognitive arousal in individuals with insomnia compared to “good sleepers” [54, 55].

## Methods

This study was a two-arm, parallel-group, single-blind, superiority randomized controlled trial. Methodologies are summarized below, with the full protocol published [56]. The trial was prospectively registered with the Australian New Zealand Clinical Trial Registry (ACTRN12619001636145). This paper follows recommendations from the Consolidated Standards of Reporting Trials (CONSORT) 2010 guidelines, the CONSORT checklist (see Supplementary material), the CONSORT Social and Psychological Interventions checklist (CONSORT-SPI), and the CONSORT Patient-Reported Outcomes checklist (CONSORT-PRO) [57–60].

### Participants

Inclusion criteria were: (1) individuals aged ≥18 years, (2) ability to communicate in English; (3) regular access to internet, smartphone, and email, and (4) score ≥10 on the Insomnia Severity Index (ISI) [61].

Exclusion criteria were: (1) shift work (fixed night shift working between 12am-5am and rotating work schedules involving night shift) during the study period, (2) significant symptoms of the following sleep disorders based on Duke Structured Clinical Interview for Sleep Disorders (DSISD) [62]: sleep apnea (apnea–hypopnea index > 15 and not or inadequately treated), periodic limb movement disorder (arousal index > 15), restless legs syndrome (3 times/week, at least 1 month), circadian rhythm sleep–wake disorders including irregular, non-24-hour, advanced (habitual bedtime earlier than 08:00 pm and habitual wake time earlier than 04:00 am, occasional deviation allowed), and delayed (habitual bedtime later than 03:00 am and habitual wake time later than 11:00 am, occasional deviation allowed) sleep phase types; (3) at risk determined by individuals who select either moderate or high on the suicidal ideation item on the baseline questionnaire, and (4) using Fitbit or Dreem in the month prior to participation.

### Randomization and blindings

Eligible participants were randomized 1:1 to Intervention or Control groups using permuted block randomization generated in advance, in block sizes of 4, 6, and 8. Random seeds were generated to assure allocation concealment and pre-guessing of the allocation sequence at the end of each block. Randomization was stratified by baseline ISI (≤14 and ≥ 15). The randomization scheme was generated and setup in REDCap [63] by a member of the research team (JFW) who (1) was not involved in recruitment or delivery of the study intervention and (2) was not one of the Principal Investigators. Follow-up study measures were either self-completed or conducted by research staff who were blinded to the group. Participants were not blinded but asked to withhold group allocation from research staff during the follow-up study measures.

### Procedures

This study was approved by Monash University Human Research Ethics Committee and was promoted as the “Novel Insomnia Treatment Experiment (NITE).” It was advertised at the Monash University Healthy Sleep Clinic (Victoria, Australia), online (e.g. relevant online forums, social media, websites, podcasts), and in public spaces (e.g. notice boards).

Interested participants provided informed consent online, and then completed a telephone screening interview to determine eligibility. Eligible participants were randomized at the end of the call. Online questionnaires were administered via REDCap (Baseline) and post-intervention (Post). All participants completed two meetings (in-person or video conference), at Baseline for orientation (~30 minutes), and 1 week later for the intervention content (~60 minutes for the Intervention and ~30 minutes for Control groups). All participants received four phone calls: (1) the screening call, (2) check-in calls 1 and 4 weeks after baseline (~10 minutes each) to encourage adherence and troubleshooting, (3) a final telephone call made by a researcher blinded to group allocation at post-intervention to administer the DSISD Insomnia module (~10 minutes).

Following the final assessment and return of research equipment, participants received up to 70 AUD in a gift voucher as a token of appreciation for participation. The gift voucher amount was calculated based on 10 AUD for each week the Fitbit was used for seven nights, and the sleep diary was completed for five nights, up to 50 AUD for 5 weeks of participation. All participants received 20 AUD for completing online questionnaires.

### Interventions

Interventions were fully manualized and scripted. All intervention sessions were recorded, and an independent rater (NQ) assessed fidelity on 10% of randomly selected participants, showing 100% fidelity for both intervention and control groups. Participants received the other group’s materials following the completion of the final assessment.

### The intervention group

Participants in the Intervention group received all following components:

1. An individual session conducted by a provisional psychologist (MAS), under the supervision of a Clinical Psychologist (BB), that covered: (1) the differences between sleep variables measured using self-report and sleep wearables, (2) the nature of sleep–wake state discrepancy and its relevance to insomnia, and (3) interpretation of sleep–wake state discrepancy using participants’ own baseline sleep data (measured via Fitbit, Dreem, and sleep diary).
2. Access to daily Fitbit sleep data through the Fitbit app, including TST, sleep start time, and wake time.
3. An illustrated report emailed weekly throughout participation (see in Protocol Supplement, OSF DOI: 10.17605/OSF.IO/T2YJS), which included both daily and weekly summaries of: (1) TST (sleep diary and Fitbit), (2) sleep efficiency (sleep diary and Fitbit), (3) SOL (sleep diary and Dreem), (4) WASO (sleep diary and Dreem) and (5) sleep stages (Dreem); sleep diary variables were either compared with Fitbit (i.e. TST and SE) or Dreem (i.e. SOL and WASO), and never both at the same time to avoid confusion. Data from two sources were plotted together (e.g. sleep diary and Fitbit TST), and these figures were accompanied by interpretations of discrepancies and information on how to use this information for managing concerns about sleep. These reports were automatically generated using an R-script that fetched data from Fitbit and Dreem servers via application programming interfaces (i.e. API).

### The control group

To control for nonspecific factors such as receiving sleep information and spending time with a researcher, the Control group was instructed to use Fitbit and Dreem without feedback about recorded sensor-based sleep throughout participation (e.g. sleep information was hidden on the Fitbit app). The Control group received a sleep education session conducted by the provisional psychologist (MAS) that covered: (1) psychoeducation about sleep (e.g. information about the function of sleep, sleep cycles, sleep stages, the homeostatic sleep drive, and circadian rhythms), and (2) sleep hygiene (e.g. healthy sleep habits regarding light, body temperature, physical activity, noise, food, caffeine, alcohol, and bedding).

### Measures

Selected measures summarized below were well validated. Details and psychometric properties of each measure are in the Protocol [56].

### Self-reported sleep

Self-reported sleep (e.g. sleep duration, onset latency, WASO) was measured using a daily sleep diary, adapted from the Consensus Sleep Diary [64]. The sleep diary collected information about experiences over the previous day and night (e.g. sleep timing, number of nighttime awakenings, naps, caffeine consumption, and medication for sleep).

### Sleep wearables

Participants wore the Fitbit Alta HR (Fitbit Inc., 2017 release) on their non-dominant hands. The Fitbit Alta HR has been found to provide equivalent estimates to research-grade actigraphy for sleep parameters such as TST, sleep latency, sleep efficiency and WASO, in and outside of the laboratory [65, 66]. However, like other actigraphy devices, compared to PSG, Fitbit has the limitations of low specificity and has been shown to overestimate TST and SE in individuals with insomnia [66, 67].

Participants were given a Dreem headband (Rhythm SAS., version 1) to wear on their head while sleeping. Compared to PSG, Dreem had high concordance for raw signal detection with 83.5% accuracy for overall scoring across five stages (i.e. all sleep stages and wake) and 74.0% accuracy with wake epochs alone (i.e. specificity) [68]. Due to discomfort and participant hesitancy associated with the headband reported by participants in the early phase of the trial, the protocol was revised, such that the use of Dreem was made an optional component after two participants were enrolled.

### Primary outcome

The primary outcome was scored in the 7-item ISI [61], with higher total scores indicating greater insomnia symptom severity. A score of 0–7 indicates no clinical insomnia symptoms; 8–14 sub-threshold insomnia; 15–21 moderate clinical insomnia; and 22–29 severe clinical insomnia [61]. Using 10 as a cut-off has demonstrated 86.1% sensitivity and 87.7% specificity for detecting clinically significant insomnia in a community sample [69]. In this study, Cronbach’s *α* for the ISI was 0.66 at Baseline (lower as only those ≥10 in the ISI distribution were included) and 0.75 at Post; Cronbach’s *α* for all other standardized questionnaire measures ranged 0.76–0.94 for baseline and 0.85–0.94 for Post.

## Secondary Outcomes

Secondary outcomes included Patient-Reported Outcome Measurement System (PROMIS; short-forms) Sleep Disturbance [70], PROMIS SRI [70], Epworth Sleepiness Scale [71]; Fatigue Severity Scale [72], and PROMIS Anxiety and Depression [73]. All PROMIS measures generated T scores, with a population mean of 50 and a standard deviation of 10.

### Exploratory outcomes

Exploratory outcomes included (1) sleep–wake state discrepancy parameters: TST discrepancies were calculated for diary-Fitbit differences (sleep diary minus Fitbit) and diary-Dreem differences (sleep diary minus Dreem); SOL and WASO differences were calculated for diary-Dreem differences (sleep diary minus Dreem), (2) the 16-item Dysfunctional Beliefs and Attitudes about Sleep [74], and (3) PSA Scale [55].

### Other measures

The insomnia module of the DSISD [62] was used to identify rates of Diagnostic and Statistical Manual of Mental Disorders Fifth Edition (DSM-5) insomnia disorder. All telephone interviews were recorded and assessed for reliability by an independent rater (NQ) with discrepancies reconciled via discussion. At Baseline, demographics, mental and physical health history, and medication use were measured through questionnaires, and perceived credibility and expectancy of treatment were measured using the Credibility Expectancy Questionnaire [75]. The Credibility Expectancy Questionnaire was collected after randomization and after participants were explained what their intervention entails; the questionnaire included questions such as: “At this point, how successful do you think this program will be at enhancing your sleep?” and “By the end of the program, how much enhancement in your sleep quality do you think will occur?” Questions related to perceived helpfulness and feedback on each intervention component, as well as the sleep wearable devices were collected post-intervention. Due to ongoing data collection during the COVID-19 pandemic, questions related to personal experiences with coronavirus disease 2019 (COVID-19) and potential impact on participation were asked of participants who completed assessments after March 2020.

Adverse effects were monitored during each participant contact throughout the research project, at all check-in phone calls, and via post-intervention questionnaire. Participants were also instructed to contact the research team immediately if they experienced unwanted adverse effects during participation.

### Statistical analysis

*A* priori power analyses were carried out for comparisons between the intervention and control groups. Assuming an attrition rate of 20%, randomizing 90 participants in total would provide power of 80% (two-tailed *a* = 0.05) to detect a medium effect size (*d* = 0.6). This effect size was based on previous behavioral interventions addressing discrepancies in self-report versus objective sleep information [44, 45].

All analyses were conducted on an intention-to-treat basis and carried out using R 4.2.0 [76]. Descriptive statistics were used to characterize demographic characteristics, rates of missing data, and all outcomes. Baseline group differences were tested using *t*-tests or *χ*^*2*^ tests.

To examine group differences in primary and secondary outcomes, separate multiple regression models were carried out. Each outcome at Post was the dependent variable, the outcome at Baseline and stratification factors were included as covariates, and intervention group was entered as the focal predictor. Following the American Statistician Association’s recommendation [77], we did not base conclusions on whether a *P*-value passes a specific threshold, but present effect sizes (ES) and 95% CI along with *P*-values for group differences. ES between groups at each time point were adjusted, mean differences, standardized by the residual variance, akin to comparing within-person effects between groups. An effect size of ~0.2, ~0.5, and ~0.8 were considered small, medium, and large.

Missing data for regression analyses were addressed using multiple imputations through chained equations with a fully conditional specification [78, 79], and all findings from regression analyses presented below are based on multiple imputations. The multiple imputation model included data from all randomized participants and 53 variables. For each variable being imputed, included predictors were: (1) the same variable from the other time point, (2) related primary and secondary outcome variables at the same time point, (3) age and sex, and (4) group allocation. Multiple imputations was conducted using the *mice* package [80] with 50 maximum iterations, and 100 multiply imputed datasets were generated. Sensitivity analyses were conducted using only participants with complete data on each outcome, and all findings are consistent with those based on multiple imputations.

## Results

**Figure 1.**
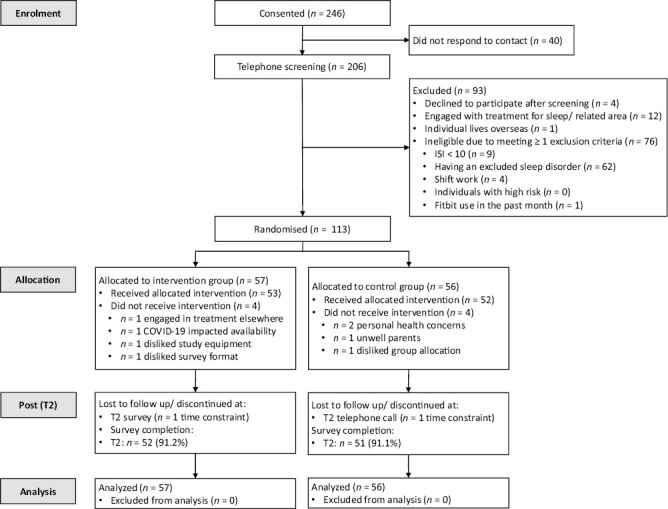
CONSORT diagram for participant flow.

Between February 7, 2020 and August 18, 2021, 206 participants gave informed consent and were screened for eligibility (see participant flow in Figure 1). A total of 113 participants were randomized to the Intervention group (*n* = 57) and control group (*n* = 56). The trial stopped due to the achievement of recruitment rates. Rates of missing data were low: among the participants randomized, 8 (7.1%) participants dropped out before commencing interventions, 105 participants (92.9%) received their allocated intervention, and 103 (91.2%) completed surveys at T2 (post-intervention). All participants who started interventions provided data on sleep diary and Fitbit: the percentages of daily sleep diary and Fitbit data throughout the 35-day protocol were 29.27 days (83.6%) and 32.04 days (91.5%), respectively. A total of 84 participants (80% of those who started the intervention) used Dreem for an average of 18.62 days (53.2% of participation days). Compared to those who provided data on Dreem, those who did not scored higher on anxiety symptoms at Baseline (*p* = .01, *d* = 0.62) but all other baseline characteristics, including all demographics and self-reported questionnaire and sleep diary variables were not notably different between those who did and did not provide Dreem data. Analyses below include all 113 randomized participants.

### Baseline characteristics

Participants (age *M*±*SD* = 47.96 ± 14.56 years) were mostly female (64.6%), white (82.9%), on average scored 17.24 (SD = 3.77; moderate insomnia severity) on the ISI, and 96.5% met DSM-5 diagnostic criteria for Insomnia Disorder. At Baseline, compared to Fitbit, participants self-reported lower average TST (by 38.79 minutes); compared to Dreem, participants self-reported lower TST (by 20.81 minutes), longer SOL (by 20.00 minutes), and greater WASO (by 18.46 minutes). Baseline characteristics, including credibility and expectancy of the intervention, were, in general, comparable between the Intervention and Control groups (d ranges 0.02–0.37 for continuous variables). Characteristics of participants are summarized in Table 1.

**Table 1.** Participant Characteristics at Baseline

	All (*N* = 113)	Control (*n* = 56)	Intervention (*n* = 57)
**Age in years, *M* (SD)**	47.96 (14.56)	48.77 (14.88)	47.17 (14.32)
***Sex***			
** Female, *n (%)***	73 (64.6)	38 (67.9)	35 (61.4)
** Male, *n (%)***	39 (34.5)	17 (30.4)	22 (38.6)
** Intersex, *n (%)***	1 (0.9)	1 (1.7)	0 (0.0)
***Ethnicity***			
** White, *n (%)***	92 (82.9)	43 (79.6)	49 (86.0)
** Asian, *n (%)***	15 (13.5)	9 (16.7)	6 (10.5)
** Black, *n (%)***	2 (1.8)	0 (0.0)	2 (3.5)
** Other, *n (%)***	2 (1.8)	2 (3.7)	0 (0.0)
***Relationship status***			
** Partnered, *n (%)***	73 (65.8)	31 (57.4)	42 (73.7)
** Not Partnered, *n (%)***	38 (34.2)	23 (42.6)	15 (26.3)
***Employment***			
** Full-time, *n (%)***	43 (39.4)	21 (38.9)	22 (40.0)
** Part-time, *n (%)***	30 (27.5)	10 (18.5)	20 (36.4)
** Unemployed, *n (%)***	36 (33.0)	23 (42.6)	13 (23.6)
***Primary*** ***outcome***			
**Insomnia severity index, *M (SD)***	17.24 (3.77)	17.41 (3.56)	17.07 (3.99)
***Secondary*** ***outcomes***			
**PROMIS sleep disturbance, *M (*SD)**	61.52 (5.68)	61.66 (6.05)	61.39 (5.35)
**PROMIS sleep-related impairment, *M (*SD)**	59.68 (6.66)	61.01 (5.87)	58.42 (7.16)
**PROMIS depression, *M (*SD)**	53.87 (7.77)	54.74 (8.52)	53.06 (6.98)
** PROMIS anxiety, *M (*SD)**	54.79 (8.48)	55.30 (8.38)	54.31 (8.61)
**Epworth sleepiness scale, *M (*SD)**	6.22 (4.52)	6.67 (4.29)	5.80 (4.73)
**Fatigue severity scale, *M (*SD)**	38.29 (11.73)	40.33 (11.95)	36.39 (11.30)
***Exploratory outcomes***			
** DBAS**	5.14 (1.53)	5.07 (1.52)	5.21 (1.55)
**Pre-sleep arousal scale (somatic), *M (*SD)**	12.17 (4.27)	12.46 (4.65)	11.89 (3.89)
**Pre-sleep arousal scale (cognitive), *M (*SD)**	21.94 (6.30)	21.60 (6.16)	22.28 (6.47)
**Pre-sleep arousal scale (total), *M (*SD)**	34.11 (9.05)	34.06 (9.35)	34.17 (8.84)
***Sleep-diary measures***			
**Total sleep time (hh:mm), *M (*SD)**	6:19 (1:09)	6:16 (1:03)	6:22 (1:16)
**Sleep onset latency (min), *M (*SD)**	47.41 (39.55)	46.92 (36.44)	47.89 (42.72)
**Wake after sleep onset (min), *M (*SD)**	42.29 (34.08)	46.27 (33.47)	38.38 (34.54)
***Fitbit measure***			
**Total sleep time (hh:mm), *M (*SD)**	6:53 (0:57)	6:56 (0:53)	6:50 (1:01)
***Dreem measures***			
**Total sleep time (hh:mm), *M (*SD)**	6:34 (1:10)	6:44 (1:04)	6:24 (1:16)
**Sleep onset latency (min), *M (*SD)**	22.54 (14.85)	22.22 (13.04)	22.85 (16.55)
**Wake after sleep onset (min), *M (*SD)**	57.36 (48.33)	53.97 (44.59)	60.58 (51.99)
***Discrepancy (sleep diary–Fitbit)***			
**Total sleep time (min), *M (*SD)**	−38.79 (70.46)	−45.30 (67.50)	−32.39 (73.32)
***Discrepancy (sleep diary–Dreem)***			
**Total sleep time (min), *M (*SD)**	−20.81 (95.06)	−17.91 (92.79)	−23.55 (98.30)
**leep onset latency (min), *M (*SD)**	20.00 (35.34)	13.36 (21.57)	26.30 (44.05)
**Wake after sleep onset (min), *M (*SD)**	−18.46 (52.52)	−15.39 (49.00)	−21.37 (56.13)
***Other Variables***			
**CEQ (credibility), *M (*SD)**	6.25 (1.23)	6.45 (1.18)	6.04 (1.25)
**CEQ (expectancy), *M (*SD)**	0.00 (0.93)	0.14 (0.84)	−0.14 (0.99)

*M* (mean) and SD (standard deviation) are presented for continuous variables, *n* (*%*) are presented for categorical variables. Discrepancy = difference between two measures, for example, sleep diary minus Dreem variables. DBAS = Dysfunctional Beliefs and Attitudes about Sleep (16-item version). CEQ = Client Expectancy Questionnaire. The Control and Intervention groups are overall comparable on all above variables, (effect-size for continuous variables range 0.02–0.37).

### Post-intervention group differences

**Figure 2.**
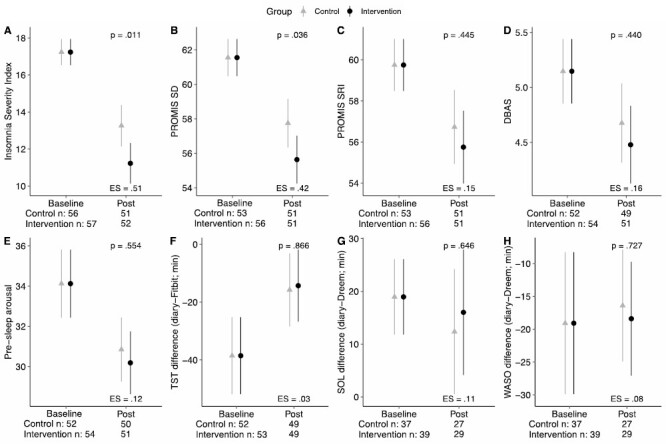
Model estimated means and 95% confidence intervals of Insomnia Severity Index (ISI), PROMIS Sleep Disturbance (SD), PROMIS SRI, Dysfunctional Beliefs and Attitudes about Sleep (DBAS), PSA, TST difference (sleep diary minus Fitbit), SOL difference (sleep diary–Dreem), and WASO difference (sleep diary–Dreem) at Baseline and Post-Intervention (Post).

Findings from the multiple regression models examining differences in post-intervention outcomes between groups are summarized in Table 2 and visually displayed in Figure 2.

**Table 2.** Summary From Multiple Regression Models Based on Multiple Imputation Examining Group Differences in Post-Intervention Outcomes Adjusting for Baseline Values

	Control (*n* = 56)	Intervention (*n* = 57)	Group Differences
*Primary outcome*			
Insomnia severity index	13.26 [12.15, 14.37]	11.23 [10.14, 12.32]	−2.03 [−3.59, −0.47], 0.011*, −0.51
*Secondary outcomes*			
PROMIS sleep disturbance	57.75 [56.35, 59.15]	55.63 [54.25, 57.02]	−2.12 [−4.10, −0.15], 0.036*, −0.42
PROMIS sleep-related impairment	56.73 [54.94, 58.52]	55.75 [53.98, 57.53]	−0.99 [−3.54, 1.57], 0.445, −0.15
PROMIS depression	52.18 [50.18, 54.17]	52.69 [50.68, 54.70]	0.53 [−2.31, 3.36], 0.713, 0.07
PROMIS anxiety	52.86 [51.14, 54.58]	53.90 [52.17, 55.63]	1.05 [−1.39, 3.48], 0.397, 0.17
Epworth sleepiness scale	5.66 [5.03, 6.30]	5.40 [4.78, 6.02]	−0.26 [−1.15, 0.62], 0.558, −0.12
Fatigue severity scale	37.42 [35.25, 39.60]	35.53 [33.37, 37.68]	−1.90 [−4.98, 1.19], 0.225, −0.24
*Exploratory outcomes*			
DBAS	4.68 [4.31, 5.04]	4.48 [4.12, 4.83]	−0.20 [−0.70, 0.31], 0.440, −0.16
Pre-sleep arousal scale (somatic)	11.62 [10.85, 12.39]	11.36 [10.59, 12.14]	−0.26 [−1.35, 0.84], 0.641, −0.10
Pre-sleep arousal scale (cognitive)	19.23 [17.85, 20.60]	18.83 [17.49, 20.17]	−0.39 [−2.31, 1.52], 0.683, −0.08
Pre-sleep arousal scale (total)	30.85 [29.26, 32.44]	30.19 [28.63, 31.75]	−0.66 [−2.88, 1.55], 0.554, −0.12
*Sleep diary measures*			
Total sleep time (hh:mm)	06:36 [06:23, 06:49]	06:33 [06:20, 06:46]	−2.85 [−21.18, 15.49], 0.759, −0.06
Sleep onset latency (min)	33.85 [25.01, 42.68]	38.83 [29.90, 47.77]	4.94 [−7.60, 17.48], 0.436, 0.16
Wake after sleep onset (min)	37.01 [29.12, 44.89]	40.87 [33.01, 48.72]	3.85 [−7.30, 15.00], 0.494, 0.14
*Fitbit measure*			
Total sleep time (hh:mm)	06:50 [06:35, 07:06]	06:47 [06:32, 07:02]	−3.14 [−24.60, 18.32], 0.772, −0.06
*Dreem measures*			
Total sleep time (hh:mm)	06:31 [06:12, 6:51]	06:32 [06:14, 06:51]	0.95 [−24.29, 26.18], 0.94, 0.02
Sleep onset latency (min)	24.49 [20.69, 28.28]	23.28 [19.54, 27.01]	−1.22 [−6.16, 3.73], 0.625, −0.11
Wake after sleep onset (min)	49.84 [42.28, 57.39]	50.24 [43.12, 57.35]	0.40 [−9.09, 9.90], 0.933, 0.02
*Discrepancy (sleep diary– Fitbit)*			
Total sleep time (min)	−15.77 [−28.33, −3.21], 0.014*	−14.30 [−26.74, −1.86], 0.025*	1.51 [−16.23, 19.24], 0.866, 0.03
*Discrepancy (sleep diary–Dreem)*			
Total sleep time (min)	12.40 [−6.85, 31.66], 0.203	13.53 [−5.37, 32.43], 0.158	1.14 [−24.06, 26.33], 0.929, 0.02
Sleep onset latency (min)	12.36 [0.51, 24.20], 0.041*	16.03 [4.16, 27.89], 0.009**	3.64 [−12.09, 19.38], 0.646, 0.11
Wake after sleep onset (min)	−16.40 [−24.91, −7.89], <.001***	−18.40 [−27.07, −9.74], *p* < .001***	−1.99 [−13.35, 9.36], 0.727, −0.08

In this table, model estimated mean [95% confidence intervals] are shown for all variables in Control and Intervention groups at post-intervention time point, adjusting for baseline values of each variable. For Group differences, model estimated mean [95% confidence interval], *P*-values, and ES are presented. ES between groups were adjusted, standardized mean differences, standardized by residual variance, comparable to within-person effects. Effect sizes of ~0.2, ~0.5, and ~0.8 are considered small, medium, and large. Discrepancy = difference between two measures, for example, sleep diary minus Dreem variables. DBAS = Dysfunctional Beliefs and Attitudes about Sleep. CEQ = Client Expectancy Questionnaire. ***p < .001, **p < .01, *p < .05.

Adjusting for baseline ISI scores, the Intervention group showed lower ISI (*p* = .011, ES = 0.51) compared to the Control group at Post, suggesting a greater reduction in insomnia symptom severity from Baseline to Post in the Intervention group. Similarly, the Intervention group also had lower PROMIS Sleep Disturbance at Post, after controlling for baseline scores (*p* = .036, ES = 0.42). At Post, 58.8% and 78.4% of those allocated to Intervention and Control group met criteria for DSM-5 Insomnia Disorder, respectively (*p* = .054).

All other secondary and exploratory variables, including PROMIS SRI, Epworth Sleepiness Scale, Fatigue Severity Scale, PROMIS Anxiety, PROMIS Depression, DBAS, PSAS, and all sleep–wake state discrepancy variables, did not show notable differences between the Intervention and Control groups post-intervention (*P*-values range.23–.93, ES range 0.02–0.24).

### Within-group differences across intervention

To describe patterns of changes within either group, paired-sample *t*-tests were used to explore the differences between Baseline and Post within each group. These exploratory analyses showed that both Intervention and Control groups had similar patterns of change (see Supplementary Table S1 and S2 in the Supplement), including: (1) improvements in insomnia-related domains such as the ISI, PROMIS Sleep Disturbance and SRI, DBAS and cognitive PSA (*d* ranges 0.32–1.29), (2) reduced sleep duration discrepancy (*d* ranges 0.38-0.56), and (3) little changes in Fitbit or Dreem measured TST (*d* ranges 0.06–0.13), or diary-Dreem discrepancy on SOL or WASO (*d* ranges 0.04–0.2). The Control (but not the Intervention) group had a small reduction in symptoms of depression (*d* = 0.29), anxiety (*d* = 0.42), diary measured SOL (*d* = 0.37) and WASO (*d* = 0.28).

### COVID-19 impact on participation

Of the 99 participants who completed the COVID-19 impact questionnaire, 68 (68.7%) reported that none of their participation was affected by the pandemic, with the remaining affected to some degree during their participation. No participant tested positive for COVID-19 during their participation. Participants reported that the pandemic had a negative impact on their mental health (49.5%), physical health (28.3%), relationships with others (44.4%), employment (29.3%), and finances (25.3%). Most participants (76.8%) reported that the pandemic had no impact on their ability to use strategies/ resources provided by the study.

### Feedback on study components

Of the 51 participants allocated to the Intervention group and completed post-intervention questionnaires, most found the following helpful: the initial meeting to learn about sleep and sleep discrepancy (96.1%), check-in calls with the research team (94.1%), gaining greater insight into sleep through comparisons between own reported and recorded sensor-based sleep data in the weekly reports (92.2%), receiving daily information about sleep through the Fitbit (84.3%). Qualitative feedback from the Intervention group showed that participants found information provided in the study helpful in “reducing sleep anxiety/ worry,” and “sleep may not be as bad as is recalled,” but many expressed that “this knowledge did not help change sleep behaviors or quality.”

Of the 49 participants that completed the study feedback scale from the Control group at post-intervention, 44 (89.8%), and 39 (79.6%) of participants found the initial meeting to learn about sleep and the check-in calls with the research team to be helpful, respectively.

### Adverse events

Throughout participation, 37 participants reported during study contact and check-in calls that they had discomfort in using the Dreem headband. Dreem discomfort was also reported by 67 participants in the post-intervention questionnaire, including Dreem impacting sleep (e.g. movement due to the loose fit, pressure due to sleep position) and/or causing tenderness or headaches. During the study, modifications were made to address some of these concerns (e.g. using elastic tighteners to improve fit and having breaks between uses); participants were free to discontinue using Dreem if they chose to.

Additionally, eight participants reported during study contact and check-in calls, that they had discomfort in using Fitbit. Fitbit discomfort was also reported by nine participants in the post-intervention questionnaire, including lighting on Fitbit disrupting sleep, loose fit, and/or skin irritation. During the study, modifications were made to address these concerns (e.g. providing a smaller Fitbit band, covering the device screen to reduce light); participants were free to discontinue using Fitbit if they chose to.

## Discussion

This randomized-controlled trial investigated whether providing individuals with insomnia symptoms support for interpretation of recorded sensor-based sleep data improved insomnia symptom severity compared to sleep hygiene and education control. Overall, the intervention was feasible as indicated by the high satisfaction and low dropout. After the intervention, the Intervention (vs. Control) group had lower insomnia symptom severity and sleep disturbance (medium ES), and lower rates of Insomnia Disorder. However, the two groups did not meaningfully differ on all other secondary and exploratory outcomes at post-intervention, including SRI, PSA, dysfunctional beliefs and attitudes about sleep, symptoms of depression and anxiety, and sleep–wake state discrepancy metrics (TST, SOL, WASO). Within-group changes from pre- and post-intervention were more similar than different in the Intervention and Control groups: both showed improvements in some sleep and insomnia domains but not others.

Group differences in post-intervention insomnia symptoms and sleep disturbance are consistent with Tang and Harvey’s [45] study comparing groups who were taught or not taught about sleep–wake state discrepancy. This suggests that providing feedback on wearable-measured sleep with education and guidance on sleep–wake state discrepancy has the potential to improve symptoms of insomnia. However, compared to the average ES of Cognitive Behavioral Therapy for Insomnia (CBT-I) relative to a control group, these effects are smaller (e.g. on ISI, 0.85 for CBT-I vs 0.51 in this study) [81]. The mean insomnia severity reduction in the intervention group for this study was 6.11 (35.79%), which is a smaller mean change compared with other research exploring insomnia symptom reductions following CBT-I. For example, a reduction of 7.59 (40.72%) for in-person CBT-I in a study exploring CBT-I modality differences [82], 9.09 (67.99%) in a study exploring insomnia in older adults [13], and 10.57 (57.10%) in a study of individuals with chronic insomnia [83]. The lack of meaningful group differences in other sleep and mental health-related domains further suggests that although discussing sleep–wake state discrepancy improved insomnia symptoms, the benefits in other related domains are likely comparable to that of sleep education and sleep hygiene.

There are a few possible explanations for the modest effects of the Intervention group in this study:

(1) Although sleep education and hygiene are often used as the control group in cognitive behavioral sleep interventions, it is consistently shown to have benefits to sleep [84]. In the sleep education and hygiene condition, we observed significant pre-post intervention changes in self-reported TST, self-reported SOL, TST sleep–wake state discrepancy, insomnia symptom severity, sleep disturbance, SRI, dysfunctional beliefs and attitudes about sleep, PSA, depression, and anxiety. This is consistent with findings from Chung et. al.’s [84] meta-analysis and systematic review that showed improved self-reported TST, self-reported SOL, and insomnia severity following participation in a sleep hygiene protocol.
(2) The Intervention group received education and guidance on sleep–wake state discrepancy but did not receive intervention targeting known perpetuating factors of insomnia, such as systematically addressing different types of sleep-related beliefs and strategies to manage PSA. This is consistent with the little group difference in changes of DBAS and PSA in this study. In the absence of these cognitive components, receiving wearable measured sleep alone might have perpetuated sleep-related anxiety. Terms such as “orthosomnia” [85, 86] are used to describe the perfectionistic quest for better sleep that can occur when individuals have access to sleep data from wearable devices. Furthermore, it is possible that some of the sensor-based sleep nights throughout participation may be shorter or worse than perceived, and receiving “negative” feedback about sleep has been shown to be associated with monitoring of sleep-related threats and negative thoughts [32, 87].

Overall, there were no meaningful group differences post-intervention in any of the sleep–wake state discrepancy parameters (TST, SOL, and WASO), suggesting that providing feedback and guidance on wearable-measured sleep (i.e. directly targeting sleep–wake state discrepancy) did not alter sleep perception more than sleep education and hygiene (i.e. no content targeting sleep–wake state discrepancy). It is possible that mechanisms of sleep–wake state discrepancy not addressed in this study played an important role in its maintenance. Emerging research highlights the importance of physiological aspects such as the mixture of wake and sleep activity during sleep and the sensitivity to sleep fragmentation [35–38] being highly relevant to individuals’ experience of sleep–wake state. It is possible that these physiological processes were not readily modified by the cognitively focused intervention attempted in this study. It is also possible that wearable-measured sleep may not have been perceived as being accurate by participants, or that some may have held rigid beliefs about their sleep–wake experiences; these cannot be confirmed as neither aspect was measured in this study. Nevertheless, both groups showed reduced sleep–wake state discrepancy duration with small to medium ES. A possibility for this finding in both groups could be that improved sleep itself is associated with less sleep–wake state discrepancy. Therefore, as participants sleep improved throughout the intervention, their sleep–wake state discrepancy also reduced. It is worth noting that despite little group differences in sleep–wake state discrepancy, the intervention group did have a greater reduction in insomnia symptoms; although future research is needed regarding the mechanisms through which insomnia improved, it is likely changes in sleep–wake state discrepancy did not play a meaningful role.

### Limitations

Exclusion criteria of the study means that findings may not generalize to individuals with comorbid insomnia and severe physiologically based sleep disorders or those with severe psychiatric or health conditions. The sample was mostly White, in a relationship, and working full-time, further limiting findings to more diverse samples. Missing data on sleep wearable devices, especially on the EEG Dreem headband, may have impacted the quality of sleep reports that participants received. This study occurred throughout the COVID-19 pandemic, and although participants reported relatively small impact of the pandemic on their participation and overall well-being, findings may not be generalized to different societal contexts and timeframes. This manuscript did not explore physiological mechanisms of sleep–wake state discrepancy (e.g. using EEG derived from Dreem), which needs to be explored in future research. We did not use a questionnaire to systematically assess itemized adverse events, but all participants were proactively asked about adverse events at each contact. Finally, this study did not explore individual differences in responses to the intervention; it is possible that some individuals with insomnia may benefit, whilst others do not benefit from receiving sleep feedback, and factors predicting these responses require future research.

## Conclusions and Implications

Addressing sleep–wake state discrepancy by providing education and guidance on wearable measured sleep data could reduce symptoms of insomnia. However, the scope of these benefits was modest compared to sleep education and sleep hygiene, and likely inferior to that of CBT-I.

From the perspective of increasing consumer sleep wearable devices, this study underlines the need for further research in how sleep data are presented to (1) relatively healthy individuals, and (2) individuals with insomnia and sleep concerns. Findings from this study provide empirical evidence for the provision of guidance (such as those used in this study) alongside wearable sleep data to support healthy interpretation. This may be particularly important for individuals with insomnia where sleep–wake state discrepancy is especially pronounced. However, this study also highlighted that providing sleep data along with guidance of interpretation is likely not sufficient as a treatment for insomnia on its own.

From the perspective of insomnia treatment, CBT-I is an established effective approach, yet it does not traditionally incorporate recorded sensor-based sleep data. Furthermore, there are increasing numbers of individuals presenting to sleep services with their own recorded sensor-based sleep data, who have an expectation that clinicians utilize that information [45–48]. There may be potential to extend the use of sleep diaries, a core component of CBT-I, by incorporating recorded sensor-based sleep data, which could provide an opportunity to start a discussion about sleep–wake state discrepancy. Future studies need to examine how sleep–wake state discrepancy guidance using wearable devices could supplement CBT-I, and whether this supplementation could enhance the benefits of CBT-I.

## Supplementary Material

zsad167_suppl_Supplementary_Material

## Data Availability

De-identified data from this trial will be shared on reasonable request to the corresponding authors.
