# Supplementary material for: Does providing feedback and guidance on sleep perceptions using sleep wearables improve insomnia? Findings from “Novel Insomnia Treatment Experiment”: a randomized controlled trial
Source: Sleep. 2023 Jun 9;46(9):zsad167. doi: 10.1093/sleep/zsad167 (PMC10485571; doi:10.1093/sleep/zsad167)

# Providing feedback and guidance on sleep perceptions using sleep wearables improves insomnia: Findings from the randomised Novel Insomnia Treatment Experiment (“NITE”)

Marie-Antoinette Spina^1^, Thomas Andrillon^3,4^, Nina Quin^1^, Joshua F. Wiley^1^, Shantha M. W. Rajaratnam^1^, Bei Bei^1,2^

1. Turner Institute for Brain and Mental Health, School of Psychological Sciences, Faculty of Medicine, Nursing and Health Sciences, Monash University, Victoria, Australia;
2. Women’s Mental Health Service, Royal Women's Hospital, Victoria, Australia;
3. School of School of Philosophical, Historical, and International Studies, Centre for Consciousness and Contemplative Studies, Monash University, Melbourne 3168, Victoria, Australia;
4. Paris Brain Institute, Sorbonne Université, Inserm-CNRS, Paris, 75013, France.

Correspondence to: Bei Bei, DPsych(Clinical), PhD, Turner Institute for Brain and Mental Health, School of Psychological Sciences, Faculty of Medicine, Nursing and Health Sciences, Monash University, 18 Innovation Walk, Clayton Campus, Victoria 3800, Australia. [bei.bei@monash.edu](about:blank).

Table S1

*Baseline and post NITE study differences for the intervention group.*

|  | **Baseline** | **Post-Intervention** | **Change, *t*(*df*), *p*, *d*** |
| --- | --- | --- | --- |
| **Primary Outcome** | | | |
| Insomnia severity index | 17.07 (3.99) | 10.96 (4.06) | -9.30 (51), < .001, 1.29 |
| **Secondary Outcomes** | | | |
| PROMIS sleep disturbance | 61.39 (5.35) | 55.40 (6.13) | -6.85 (50), < .001, 0.96 |
| PROMIS sleep-related impairment | 58.42 (7.16) | 54.68 (8.01) | -3.30 (50), 0.002, 0.46 |
| PROMIS depression | 53.06 (6.98) | 51.73 (9.12) | -0.87 (50), 0.387, 0.12 |
| PROMIS anxiety | 54.31 (8.61) | 53.30 (8.50) | -0.70 (50), 0.486, 0.10 |
| Epworth sleepiness scale | 5.80 (4.73) | 5.10 (4.27) | -2.30 (51), 0.026, 0.32 |
| Fatigue severity scale | 36.39 (11.30) | 33.94 (10.25) | -1.78 (51), 0.081, 0.25 |
| **Exploratory Outcomes** | | | |
| DBAS | 5.21 (1.55) | 4.46 (1.57) | -3.72 (50), < .001, 0.52 |
| Pre-sleep arousal scale (somatic) | 11.89 (3.89) | 11.24 (3.84) | -1.65 (50), 0.105, 0.23 |
| Pre-sleep arousal scale (cognitive) | 22.28 (6.47) | 19.08 (6.56) | -4.97 (50), < .001, 0.70 |
| Pre-sleep arousal scale (total) | 34.17 (8.84) | 30.31 (8.39) | -4.92 (50), < .001, 0.69 |
| **Sleep Diary Measures** | | | |
| Total sleep time (hh:mm) | 06:23 (01:16) | 06:35 (01:16) | 1.97 (49), 0.054, 0.28 |
| Sleep onset latency (min) | 47.89 (42.72) | 39.15 (46.62) | -1.54 (49), 0.129, 0.22 |
| Wake after sleep onset (min) | 38.38 (34.54) | 38.78 (34.87) | 0.07 (49), 0.943, 0.01 |
| **Fitbit Measure** | | | |
| Total sleep time (hh:mm) | 06:50 (01:01) | 06:47 (01:15) | -0.79 (48), 0.432, 0.11 |
| **Dreem Measures** | | | |
| Total sleep time (hh:mm) | 06:24 (01:16) | 06:33 (01:19) | 0.37 (26), 0.714, 0.07 |
| Sleep onset latency (min) | 22.85 (16.55) | 23.03 (17.82) | 0.36 (26), 0.721, 0.07 |
| Wake after sleep onset (min) | 60.58 (51.99) | 53.07 (33.89) | -1.46 (26), 0.155, 0.28 |
| **Discrepancy (sleep diary – Fitbit)** | | | |
| Total sleep time (min) | -32.39 (73.32) | -12.75 (53.48) | 2.69 (48), 0.010, 0.38 |
| **Discrepancy (sleep diary – Dreem)** | | | |
| Total sleep time (min) | -23.55 (98.30) | 17.16 (76.58) | 2.39 (26), 0.024, 0.46 |
| Sleep onset latency (min) | 26.30 (44.05) | 17.80 (38.06) | -0.50 (26), 0.624, 0.10 |
| Wake after sleep onset (min) | -21.37 (56.13) | -21.38 (32.65) | 0.26 (26), 0.796, 0.05 |

*Note*. *M* (mean) and *SD* (standard deviation) are presented for baseline and post-intervention for those who completed the measures; for change variables the paired samples t-value (degrees of freedom; *df*)’, *p*-values, and Cohen’s *d* are presented. DBAS = Dysfunctional Beliefs and Attitudes about Sleep.

Table S2

*Baseline and post NITE study differences for the control group.*

|  | **Baseline** | **Post-Intervention** | **Change, *t*(*df*), *p*, *d*** |
| --- | --- | --- | --- |
| **Primary Outcome** | | | |
| Insomnia severity index | 17.41 (3.56) | 13.31 (4.61) | -6.58 (50), < .001, 0.92 |
| **Secondary Outcomes** | | | |
| PROMIS sleep disturbance | 61.66 (6.05) | 57.74 (6.08) | -5.30 (50), < .001, 0.74 |
| PROMIS sleep-related impairment | 61.01 (5.87) | 57.39 (7.29) | -4.09 (50), < .001, 0.57 |
| PROMIS depression | 54.74 (8.52) | 52.66 (9.28) | -2.08 (49), 0.043, 0.29 |
| PROMIS anxiety | 55.30 (8.38) | 53.04 (9.21) | -2.95 (49), 0.005, 0.42 |
| Epworth sleepiness scale | 6.67 (4.29) | 6.02 (3.92) | -1.80 (49), 0.077, 0.26 |
| Fatigue severity scale | 40.33 (11.95) | 38.53 (11.06) | -1.23 (49), 0.224, 0.17 |
| **Exploratory Outcomes** | | | |
| DBAS | 5.07 (1.52) | 4.55 (1.92) | -2.75 (48), 0.008, 0.39 |
| Pre-sleep arousal scale (somatic) | 12.46 (4.65) | 11.96 (4.55) | -1.48 (49), 0.145, 0.21 |
| Pre-sleep arousal scale (cognitive) | 21.60 (6.16) | 19.02 (6.62) | -3.82 (49), < .001, 0.54 |
| Pre-sleep arousal scale (total) | 34.06 (9.35) | 30.98 (9.41) | -3.55 (49), < .001, 0.50 |
| **Sleep Diary Measures** | | | |
| Total sleep time (hh:mm) | 06:16 (01:03) | 06:35 (01:14) | 2.99 (49), 0.004, 0.42 |
| Sleep onset latency (min) | 46.92 (36.44) | 32.69 (33.22) | -2.62 (49), 0.012, 0.37 |
| Wake after sleep onset (min) | 46.27 (33.47) | 38.73 (31.36) | -1.97 (49), 0.055, 0.28 |
| **Fitbit Measure** | | | |
| Total sleep time (hh:mm) | 06:56 (00:53) | 06:55 (00:54) | -0.43 (48), 0.670, 0.06 |
| **Dreem Measures** | | | |
| Total sleep time (hh:mm) | 06:44 (01:04) | 06:41 (01:03) | -0.66 (48), 0.514, 0.13 |
| Sleep onset latency (min) | 22.22 (13.04) | 26.75 (17.90) | 2.07 (24), 0.049, 0.41 |
| Wake after sleep onset (min) | 53.97 (44.59) | 48.83 (38.30) | -1.93 (24), 0.065, 0.39 |
| **Discrepancy (sleep diary – Fitbit)** | | | |
| Total sleep time (min) | -45.30 (67.50) | -20.59 (70.42) | 3.91 (48), < .001, 0.56 |
| **Discrepancy (sleep diary – Dreem)** | | | |
| Total sleep time (min) | -17.91 (92.79) | 12.59 (80.51) | 2.49 (24), 0.020, 0.50 |
| Sleep onset latency (min) | 13.36 (21.57) | 9.53 (31.38) | -1.01 (24), 0.324, 0.20 |
| Wake after sleep onset (min) | -15.39 (49.00) | -16.09 (36.29) | 0.22 (24), 0.828, 0.04 |

*Note*. *M* (mean) and *SD* (standard deviation) are presented for baseline and post-intervention; for change variables the t-value (degrees of freedom; *df*), *p*-values, and effect sizes are presented. DBAS = Dysfunctional Beliefs and Attitudes about Sleep.

## Feedback of Study Components: Further Qualitative Information.

Qualitative feedback from the Intervention group indicated mixed responses to recording sleep utilising Fitbit and Dreeem devices. Some individuals enjoyed being able to see their sleep data while others felt that the devices were too difficult to use. Specifically, one participant reported, “It was good getting some solid data, as opposed to just relying on inaccurate recollection” while another participant stated that they “enjoyed monitoring my sleep via the Fitbit [but] I hated doing the sleep diary”. Numerous comments from participants highlighted thoughts relating to the intervention session, again with mixed feelings about how useful the session was in terms of their sleep. For example, one participant described “while it's been interesting to learn more about sleep stages and how my sleep varies each night, I don't feel it's actively helped me to change my sleep patterns or sleep quality”, and another participant highlighted “I enjoyed my participation in this study and the devices gave me a greater understanding of my sleep pattern, although this has not improved my sleeping. My conclusion is that my sleep is not as poor as I thought but that does not make me feel better nor hopeful of feeling more rested." Interestingly, one participant described “The favourite part I like most of this experience is when I got a chance to learn from the researcher about the sleep cycle, how the brain functions, and debunk the myths about sleeping. I feel I can apply some knowledge to improve my sleep habit[s].”, and another individual stated “I did find it extremely helpful to talk about what a normal nights sleep can look like.” In terms of sleep-state discrepancy, some individuals highlighted this within the comments like this one participant who “Realised I sleep much more than I thought I did”, while another participant highlighted, “I think I have learnt that I sleep better than I thought I did, and also not to worry about my sleep as much."

Qualitative feedback from the Control group highlighted that most participants enjoyed the information session, however, there were mixed views as to whether the information was helpful for their sleep. One participant reported “The educational slide show of how the body works and how sleep works is very helpful”, while another participant stated “In terms of sleep strategies, I'm not sure that my difficulties were addressed”.


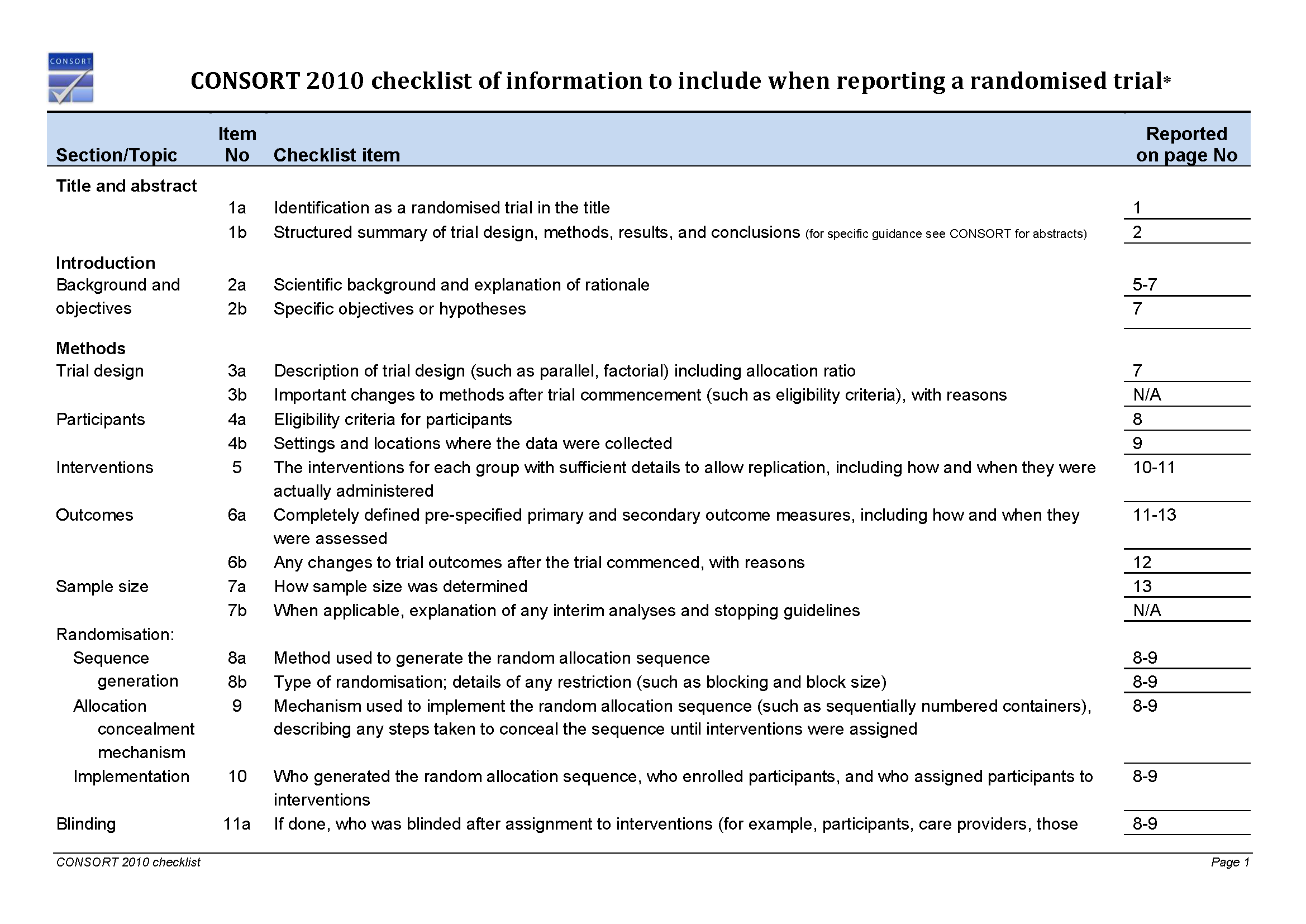


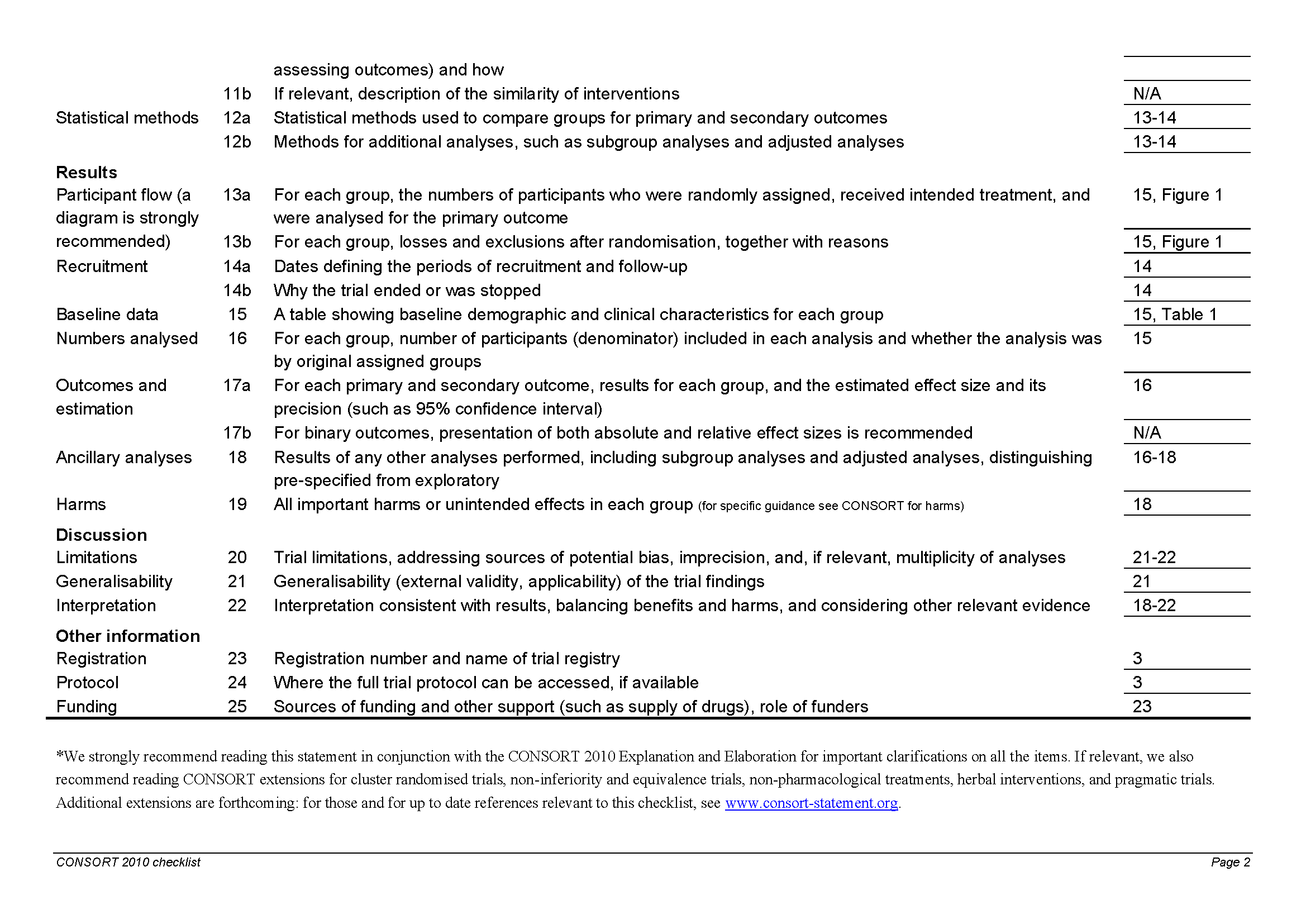

Supplement: zsad167_suppl_Supplementary_Material [file zsad167_suppl_supplementary_material.docx]
